# Supplementary material for: Validation of a hierarchical algorithm to define chronic liver disease and cirrhosis etiology in administrative healthcare data
Source: PLoS One. 2020 Feb 18;15(2):e0229218. doi: 10.1371/journal.pone.0229218 (PMC7028265; doi:10.1371/journal.pone.0229218)
Supplement: Attachment — Submitted filename: Flemming_Validation_further info Dec 13.docx [file pone.0229218.s001.docx]

**Thank you for the request for further information. Below please see our response to them. We hope we have been able to address them to the satisfaction of PLOS One.**

PONE-D-19-27866R1
Validation of a hierarchical algorithm to define chronic liver disease and cirrhosis etiology in administrative healthcare data
Dr. JA Flemming

Dear Dr. Flemming,

Thank you for submitting your manuscript entitled "Validation of a hierarchical algorithm to define chronic liver disease and cirrhosis etiology in administrative healthcare data" to PLOS ONE. Your manuscript files have been checked in-house but before we can proceed we need you to address the following issues:

1) Thank you for your response to our requests and for indicating that the data were analyzed anonymously in the ethics statement of the online submission form. At this time, we also ask that you state that the data were analyzed anonymously in the ethics statement of the Methods section in the manuscript.

Thank you for your attention to this request.

**RESPONSE: Change made. Please see revised manuscript Methods section paragraph 4.**

2) Thank you for describing the data sharing restrictions in your statement:

"The authors are affiliated with ICES and conducted the study in fulfillment of ICES’ mandate as a prescribed entity under Ontario’s Personal Health Information Protection Act. As a result, the authors were authorized, both legally and contractually, to access the data set in a more granular form than approved third party researchers would be permitted to access. The data set that approved third party researchers would be permitted to access would be adjusted to ensure the risk of re-identification of any underlying individuals is low. While data sharing agreements and privacy legislation for the province of Ontario prohibit ICES from making the data set publicly available, access may be granted to those who meet pre-specified criteria for confidential access, available at www.ices.on.ca/DAS."

At this time, please address the following:

1) Please fully disclose the method of data collection, including relevant data set names or variables, for qualified researchers to replicate your study.

**RESPONSE: Thank you. Please see additional text added to the Methods Section paragraph 4 outlining data is collected from routinely collected healthcare data. We have already outlined all the datasets used in the Methods section of the manuscript under the heading “Administrative Databases Used for Liver Disease Etiology Validation”. To confirm, the datasets for this study were 1) Registered Persons Database (RPDB); 2) Canadian Institute for Health Information Discharge Abstract Database (CIHI DAD); 3) National Ambulatory Care Reporting System (NACRS); 4) OHIP Physician Claims Database; 5) Ontario Laboratory Information System (OLIS); 6) Public Health Ontario (PHO) Viral Hepatitis data. We have numbered them now in the manuscript to make it clearer for readers.**

2) Please clarify what the qualifications are for researchers to be approved for confidential access.

**RESPONSE: Thank you. Researchers, students, policy makers or knowledge users who are affiliated with a publicly funded, not-for-profit organization and who want to obtain and analyze ICES data to answer a research question may submit a request to ICES DAS. DAS staff will contact the requestor to discuss the project’s feasibility, timeline and cost. Projects requesting access to data require the approval of a research ethics board.**

**DAS staff work with requestors to design a research-ready data extract from the ICES data repository using an algorithm that de-identifies data in a way that provides researchers with individual-level data while meeting all privacy standards. Requestors are provided with access to the linked, de-identified data extract on a secure, online research environment, where they can perform analyses and create reports. To ensure privacy policy compliance, linked data cannot be copied or removed from the environment.**

I look forward to your response and upon receiving this information I will update your Data Availability Statement on your behalf.

Your manuscript has been returned to your account. Please log on to PLOS Editorial Manager at https://www.editorialmanager.com/pone/ to access your manuscript.

Your manuscript can be found in the "Revisions Sent Back to the Author" link under the New Submissions menu. After you have made the changes requested above, please be sure to view and approve the revised PDF after rebuilding the PDF to complete the resubmission process.

Please note that these changes have been requested to comply with submission guidelines and your manuscript will *not* be sent to review until you have fully adhered to our requests. Once your paper has been seen by an Editor we may return it to you for further information or amendments.

We ask that you address this request within 21 days. If you require additional time, please email the journal office. We are happy to grant extensions of up to one month past this due date. If we have not heard from you within 21 days, your manuscript will be withdrawn from Editorial Manager.

Thank you for submitting your work to PLOS ONE.

Kind regards,

Anita Estes
PLOS ONE
